# Development and validation of the quiet quitting behavior scale: a mixed-methods study with primary healthcare workers in China

**DOI:** 10.3389/fpubh.2026.1773183

**Published:** 2026-03-12

**Authors:** Qing-lin Li, Tao Sun, Xiang Zhang

**Affiliations:** 1 Department of Health Administration, School of Medicine and Health Management, Tongji Medical College, Huazhong University of Science and Technology, Wuhan, China; 2 The Key Research Institute of Humanities and Social Science of Hubei Province, Huazhong University of Science and Technology, Wuhan, China; 3 Department of Health Policy and Management, School of Public Administration, Hangzhou Normal University, Hangzhou, China

**Keywords:** China, healthcare workers, psychometric validation, quiet quitting behavior, scale development

## Abstract

**Background:**

The growing prevalence of quiet quitting behavior (QQB) poses a significant challenge to workforce mental health and organizational sustainability. However, progress in this field has been hindered by the absence of culturally adapted and behaviorally anchored measurement instruments. This study aimed to develop a Quiet Quitting Behavior Scale (QQBS) tailored to the Chinese context and to validate the scale among primary healthcare workers.

**Method:**

The QQBS was developed and validated using an exploratory sequential mixed-methods design. The development process included item generation through grounded-theory analysis, content validation via Delphi consultation, instrument refinement through a pilot survey, and psychometric evaluation in a formal survey.

**Results:**

The final 15-item QQBS consists of two dimensions: Role Contraction and Behavioral Inertia (8 items) and Cognitive Collapse and Psychological Detachment (7 items). Content validity was established through expert review. Exploratory factor analysis identified a clear two-factor structure, which was subsequently confirmed by confirmatory factor analysis, indicating excellent model fit (CFI = 0.959, IFI = 0.960, RMSEA = 0.065). The scale showed excellent reliability, with high internal consistency (Cronbach’s *α* = 0.921 total; 0.874 and 0.900 subscales) and good split-half reliability (0.803). Evidence also supported robust convergent, discriminant, and criterion-related validity, the latter demonstrated by significant correlations with organizational citizenship behavior and turnover intention.

**Conclusion:**

The QQBS is a theoretically grounded, reliable, and valid instrument. It serves as a critical instrument for identifying QQBs among primary healthcare workers in China and demonstrates strong potential for application in other high-stress sectors.

## 1 Introduction

In contemporary workplaces, a behavior known as quiet quitting has attracted considerable scholarly attention (1, 2). This behavior refers to employees’ intentional reduction of work-related effort to the minimum level required to meet job expectations, without formally resigning (2). Understanding its emergence requires situating the behavior within a broader societal context. Contemporary society is undergoing a profound transformation, shifting from a disciplinary society to a performance-oriented society (3, 4). In this paradigmatic shift, social actors have evolved from being passively disciplined subjects to becoming self-exploiting agents of performance (4). This acceleration of modern rationality in the pursuit of maximum efficiency has given rise to a global syndrome of burnout (4). The expansion of instrumental rationality not only intensifies societal uncertainty but also triggers a systemic crisis in individual existential meaning (5). Individuals increasingly experience profound confusion and anxiety regarding role identity, personal goals, and life purpose, ultimately leading to the collapse of work-life balance (5). In response to this crisis of subjectivity, an increasing number of employees have begun to reflect on the deeper meaning of life and work, gradually seeking a balance between the two (6). A new form of occupational behavioral health—QQB—has thus emerged.

As a phenomenon, quiet quitting refers to employees’ efforts to reestablish work–life boundaries by performing only their core contractual duties while minimizing discretionary, extra-role contribution (7, 8). This phenomenon has emerged as a widespread challenge in workplaces globally (9). Gallup’s longitudinal surveys indicate that quiet quitters represent a substantial share of the workforce, with engagement levels remaining persistently low and highlighting serious organizational concerns (9, 10). More importantly, this phenomenon has shown an explosive trend in high emotional exhaustion industries, particularly in the healthcare (1, 11, 12). For example, a study in Greece reported that more than half of healthcare workers exhibited quiet quitting, with the prevalence highest among nurses (67.4%) (13). Such widespread disengagement not only undermines employees’ mental and physical health but also imposes substantial economic costs, with annual productivity losses estimated at $438 billion in annual global productivity losses (9, 14). Notably, these findings reveal a profound, persistent, and widespread dissatisfaction among the workforce. As an observable manifestation of quiet quitting, QQB warrants serious attention and a systematic response.

As an emerging concept, QQB has only recently attracted scholarly attention, beginning in 2022 (8, 15). Although several studies have examined its prevalence across diverse professions (1, 11, 12), the literature has primarily focused on conceptual definitions and antecedents. From different vantage points, scholars have conceptualized the phenomenon as a reduction in work input (15, 16), a disengaged psychological state (17, 18), or a gradual process of alienation (19). Some scholars further situate it within the context of post-pandemic labor reforms, interpreting it either as an extension of the Great Resignation (20) or as a strategic choice to counter burnout (16). However, the former distorts the factual basis underlying an individual’s decision to remain in their position, whereas the latter diminishes the proactive nature of the behavior. More fundamentally, although empirical studies have identified four driving factors—leadership failure (21–23), toxic organizational environments (21–24), stagnated growth opportunities (10, 25), and eroded work meaningfulness (22–24)—these factors, while highlighting the sources of stress, do not explain why some employees under pressure choose quiet quitting rather than experiencing burnout or leaving their jobs entirely. The root cause of this explanatory gap lies in the lack of clear operational definitions for behavioral anchoring, which leaves current explanations largely speculative.

This conceptual oversight has resulted in a systematic gap in the development of behavior-oriented scales. Existing research has examined the measurement of quiet quitting from three primary perspectives. From a psychological-state perspective, a study of Indian managers developed a unidimensional seven-item scale to assess insufficient individual work commitment, low organizational commitment, and a lack of proactive efforts to exceed job requirements (26). This scale has been preliminarily applied among healthcare workers (27). Similarly, a study of Chinese academics adapted turnover intention items to measure the phenomenon (7). From a behavioral-performance perspective, a Greek study constructed a nine-item scale capturing detachment, lack of motivation, and lack of initiative (28), which has since been applied in other populations (13, 29, 30). Finally, from a dynamic-process perspective, research on Chinese Gen Z employees conceptualized quiet quitting as an intention and measured it using a five-item scale adapted from organizational commitment (31) and retention intention (32) instruments (19). Current approaches to measuring quiet quitting are constrained by two interrelated gaps. First, scales developed in individualistic (26) or other Western contexts (28, 33) fail to capture the non-confrontational and implicit forms of resistance characteristic of collectivist cultures such as China. In China’s collectivist context (34), employees may exhibit surface-level role compliance while subtly renegotiating behavioral boundaries. For example, they may curtail proactive problem-solving or discretionary knowledge sharing. This pattern reflects a strategic form of adjustment that differs from Western contexts, where greater emphasis is placed on individual autonomy (35). Second, current tools often use psychological states as proxies, measuring constructs such as reduced work commitment (26), adapting turnover intention scales (7), or incorporating subjective states like lack of motivation (28). However, they frequently overlook observable indicators of responsibility contraction, such as the refusal to take on duties beyond formal job descriptions. Therefore, the development of a QQBS should not only be grounded in the local cultural context but also clearly define its conceptual essence.

The purpose of this study is to develop a QQBS tailored to the Chinese context and to validate it among primary healthcare workers. Primary healthcare workers were chosen for the initial validation because they represent a theoretically salient population with high emotional and workload demands, making them particularly susceptible to QQB. The QQBS provides a foundational instrument for researchers to further investigate this behavior and offers a validated instrument for assessing QQBS among primary healthcare workers in China.

## 2 Theoretical background and conceptualization

### 2.1 Theoretical foundations: deconstructing QQB through an integrated perspective

QQB is not a monolithic construct but rather a complex, behaviorally manifested adaptive strategy. Its core lies in the proactive redefinition of responsibility boundaries, characterized by the strategic limitation of effort to prescribed duties and the deliberate rejection of extra-role demands (16, 36). To conceptualize this behavior, it is essential to adopt a framework that integrates micro-level psychological mechanisms as well as macro-level philosophical and socio-structural contexts. At the psychological level, Cognitive Dissonance Theory (37–39) suggests that quiet quitting serves as a behavioral adjustment mechanism that alleviates the psychological tension (dissonance) resulting from the misalignment between employees’ negative cognitions and their continued participation in the workforce. Implicit Cognition Theory (40, 41) further explains the automatic, non-conscious processes underlying this behavior, whereby repeated negative experiences create cognitive schemas that elicit instinctive withdrawal responses when triggered by contextual cues. Beyond individual psychology, quiet quitting reflects broader existential and structural dilemmas. At a philosophical level, Marx’s theory of Labor Alienation (42) provides a critical lens for interpreting QQB, framing it not as simple apathy but as a latent form of resistance to structural alienation. When workers become alienated from the products, processes, and social relations of their labor, redefining boundaries serves as a means to reclaim a sense of agency. Complementing these, Xiang Biao’s Suspended Theory (43, 44) adds a socio-cultural dimension, conceptualizes QQB as an expression of suspended agency, wherein employees remain physically present while psychologically disengaged as they navigate structural constraints. This suspended stance embodies a tension between future aspirations and present disengagement, prompting a recalibration of personal values and work engagement as protective strategies. In summary, at the motivational level, Cognitive Dissonance Theory and Labor Alienation Theory elucidate the internal and external drivers that lead employees to initiate QQB. At the process and duration levels, Implicit Cognitive Theory and Suspension Theory explain how QQB is enacted and sustained as an automated and stable behavioral pattern.

Synthesizing these perspectives, QQB can be conceptualized as a threshold survival state and behavioral response that arises from the combined pressures of structural constraints and individual cognitive conflict. This response is characterized by defensive withdrawal and adaptive re-anchoring of meaning. Specifically, QQB comprises two interconnected manifestations: a behavioral strategy of deliberately restricting one’s role to formally prescribed duties, and a cognitive orientation characterized by psychological detachment from work.

### 2.2 Conceptual differentiation: demarcating the construct boundary

A precise conceptualization of QQB requires a clear distinction from related but distinct phenomena. It is not synonymous with work disengagement, low job involvement, or turnover intention. Importantly, it also differs from the related Chinese concepts of “lying flat” (tang ping) and “slacking off” (mo yu). QQB is uniquely characterized as proactive boundary sculpting (28, 45)—a strategic and deliberate redefinition of one’s responsibility boundaries. From the perspective of its occurrence mechanism, QQB represents an instrumental rational calculation under resource constraints, whereas lying flat signifies the structural collapse of a value-rational system (46). From the perspective of evolutionary characteristics, QQB is driven by the gradual deconstruction of psychological contracts, which differs from the situational regulation of slacking off (47). From an organizational perspective, QQB establishes a lock-in equilibrium of low input and low return by reducing emotional energy expenditure, thereby functioning as a buffer mechanism for resignation behavior. Thus, QQB represents a distinct, behaviorally grounded construct that requires a specialized approach to measurement. A detailed conceptual analysis is presented in Supplementary file 1.

## 3 Materials and methods

### 3.1 Methodology

#### 3.1.1 Design

**Figure 1:**
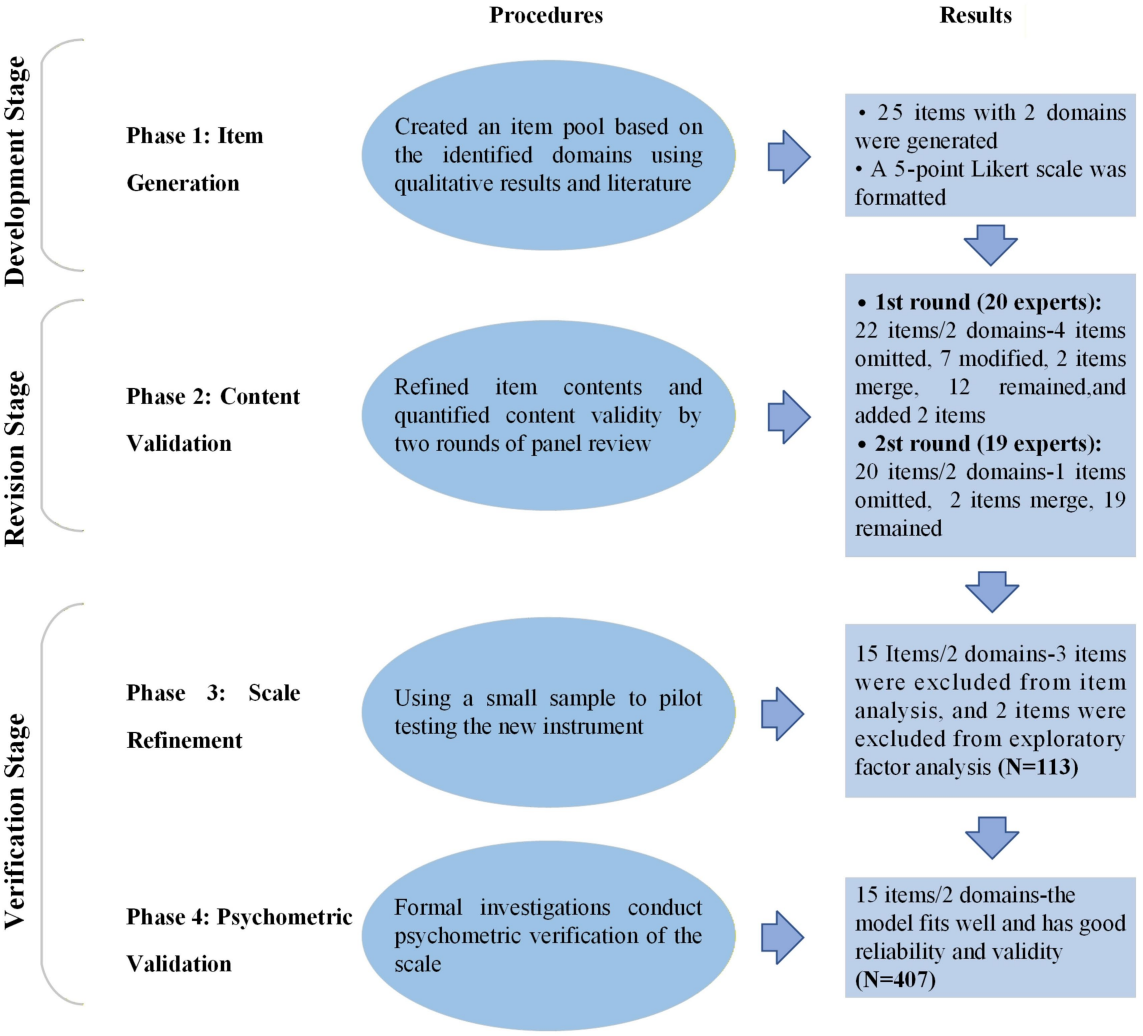
Summary of the four-phase process for developing and validating the QQBS.

An exploratory sequential mixed methods design (48) was employed to develop and validate the QQBS. The process consisted of four phases: item generation, content validation, scale refinement, and psychometric validation (see Figure 1). In the third stage and the fourth stage, we selected primary healthcare workers as the initial sample for scale validation. Given their high levels of emotional labor and workload, they frequently display distinct QQBs (11, 12, 27), thereby providing a rigorous context for evaluating the structural validity and reliability of the preliminary test scale.

#### 3.1.2 Item generation

The initial project library was developed between December 2023 and January 2024 by integrating qualitative insights from 34 in-depth interviews (see the complete interview guide in Supplementary file 2) with a comprehensive review of the existing literature on quiet quitting. The interviewees were recruited from diverse industries, including healthcare, education, technology, manufacturing, and public services. This process was designed to capture the construct’s core manifestations, primarily characterized by the redefinition of responsibility boundaries and cognitive disengagement. Over a three-month period, the research team systematically translated thematic codes and representative quotations into behavioral items, iteratively refining them to ensure they accurately captured the central concepts identified in the qualitative data. To contextualize the construct, perspectives from employees across diverse industries and organizational ranks were compared and integrated during item generation. The initial instrument consisted of 25 items measured on a 5-point Likert scale ranging from 1 (strongly disagree) to 5 (strongly agree). The two primary dimensions addressed by the scale were role contraction and behavioral inertia, and cognitive collapse and psychological detachment.

#### 3.1.3 Content validation

To ensure the content validity of the initial item pool, we conducted a two-round Delphi consultation with experts via email in May 2024. The expert panel included 9 management specialists, 7 psychology specialists, and 4 organizational behavior specialists, all of whom possessed substantial research experience or practical expertise in occupational health. The first round of expert consultation was conducted using 25 initial items. The questionnaire contained an introduction to the research background, an item evaluation module, and basic expert information, with particular emphasis on identifying items that were semantically ambiguous, culturally unsuitable, or conceptually overlapping. A five-point Likert scale (1 = very unimportant, 5 = very important) was employed to evaluate the significance of each item, while open-ended questions were included to gather specific suggestions for their improvement. Based on quantitative indices (mean importance score, coefficient of variation, and full-score ratio) as well as qualitative feedback, the items were revised, merged, or removed. The second round concentrated on validating the revised set of items. Experts were provided with a summary of the first-round results together with the modified items for re-evaluation. Based on these inputs, the final pool of scale items was established.

The reliability analysis of Delphi expert consultation primarily involves several indicators: the expert positive coefficient (E), the expert familiarity coefficient (Cs), the influence coefficient of the expert’s judgment basis (Cα), the expert authority coefficient (Cr), the coefficient of variation (CV), and the Kendall’s coefficient of concordance (W). Item screening was conducted using the full-score ratio (exclusion if < 50%), mean importance score (Mj), and coefficient of variation (CV).

#### 3.1.4 Scale refinement

The third phase of the pilot survey involving QQBS was conducted in July 2024 and aimed to perform project analysis and exploratory factor analysis (EFA). According to established methodological guidelines (49), the sample size for EFA should be at least five times the number of items included in the scale. As the scale at this stage comprised 20 items, a minimum sample size of 100 participants was required. To ensure economic diversity, we employed a stratified sampling strategy based on regional GDP. One county from each of two cities (City A and City B) in a central Chinese province was selected to represent distinct strata. Within each county, two primary medical institutions—one community health service center and one township health center—were randomly chosen. Subsequently, a cluster sample comprising all eligible healthcare workers was recruited. To enhance sample homogeneity, the study was restricted to core public primary medical institutions, including community health service center and township health center, and excluded private, specialized, or system-specific facilities, such as military clinics.

This study collected paper-based questionnaires from primary healthcare workers through on-site field investigations. The first page of each questionnaire described the study objectives and adherence to the Declaration of Helsinki, emphasizing that participation was voluntary, anonymous, and confidential, and that respondents could withdraw at any time without penalty. Completion and return of the questionnaire were considered to constitute informed consent. On average, the questionnaire required approximately 5 min to complete. Participants evaluated each item using a five-point Likert scale, ranging from 1 (strongly disagree) to 5 (strongly agree). Of the 116 questionnaires distributed, 113 were deemed valid after excluding those with logical inconsistencies, uniform responses, or more than 10% missing data, yielding an effective response rate of 97.41%. This sample satisfied the analytical requirements in terms of size and adequately reflected the structural, regional, and economic diversity of grassroots institutions.

#### 3.1.5 Psychometric validation

The fourth phase involved a large-scale investigation conducted from August to September 2024 to evaluate the QQBS from a psychometric perspective. The formal investigation was designed to conduct confirmatory factor analysis (CFA) as well as reliability and validity assessments. CFA imposes relatively stringent requirements on sample size. Based on multiple methodological recommendations (49, 50), the sample size should satisfy two criteria: it should be no fewer than 200 observations and at least 10 times the number of items on the scale. At this stage, the finalized QQBS consists of 15 items. According to the 10-to-1 rule, this corresponds to a minimum required sample size of 150. To ensure more robust parameter estimation (51, 52), we set the minimum target sample size for the formal investigation at 300. The formal survey adopted the same GDP-stratified, multistage sampling strategy as the pilot study. This approach was extended beyond the pilot phase by randomly selecting one province each from China’s eastern, central, and western regions to enhance national representativeness.

The data collection procedures and the description provided on the first page of the questionnaire were identical to those used in the pilot study. In addition to the 15 QQBS items, the questionnaire included the 4-item proactiveness subscale from the Chinese revised Organizational Citizenship Behavior Scale (Cronbach’s *α* = 0.912 in this study) (53, 54), and a single-item measure of turnover intention (55, 56). The average time required to complete the questionnaire was 8 min. All items were rated on a five-point Likert scale (1 = strongly disagree to 5 = strongly agree). We hypothesized that QQBS scores would be negatively correlated with organizational citizenship behavior and positively correlated with turnover intention, thereby providing evidence for criterion-related validity. The data cleaning procedures followed the standards established in the third stage pilot survey. After quality control, 407 valid questionnaires were retained, yielding an effective response rate of 93.14%. This sample exceeds the minimum size required for statistical analysis and is nationally representative.

#### 3.1.6 Procedural controls for common method bias

To reduce the common method bias, this study implemented three procedural control measures during the data collection stage. First, complete anonymity and strict confidentiality assurances were provided to reduce participants’ social desirability bias. Second, items from the QQBS were interspersed with those from the calibration scale to disrupt semantic proximity and minimize response pattern biases. Third, the questionnaire employed neutral instructions, explicitly stating that there were no right or wrong answers and encouraging honest responses, thereby fostering psychological separation among participants’ response roles. Collectively, these procedures were designed to systematically reduce the potential impact of common method bias at the data collection stage.

### 3.2 Data analyses

Data analysis proceeded in sequential phases aligned with the study’s exploratory mixed-methods design. Qualitative data from semi-structured interviews were analyzed using a grounded theory approach with NVivo 11.0. The qualitative research phase ensured the reliability and validity of the study through standardized procedures for data collection, systematic coding analysis, and theoretical saturation testing. First, open coding was conducted line by line to generate initial categories. These categories were then compared and aggregated into broader main categories through axial coding. Finally, selective coding was used to integrate the main categories and identify two core categories. To enhance methodological rigor, all coding procedures were performed independently by two researchers. Through open, axial, and selective coding (57), the core categories of QQB were identified, which served as the foundation for developing the initial item pool. Content validity was assessed through two rounds of Delphi expert consultations, and its quality was evaluated using multiple indicators. In general, an E-value greater than 70% indicates strong expert positivity. Expert authority is assessed using the authority coefficient (Cr), calculated as Cr = (Cs + Cα) / 2, with values of Cr ≥ 0.70 considered acceptable (58). The degree of concentration in expert opinions is measured using the mean (Mj) and the coefficient of variation (CV) of experts’ ratings on the importance of indicators. In general, an Mj value greater than 4.00 and a CV value below 0.30 are considered acceptable (59). When W > 0.40 and the χ^2^ test shows statistical significance, the degree of coordination is regarded as satisfactory (60). Item selection followed a two-step process. First, items with a full-score ratio below 50% or consistently recommended for deletion were excluded. Second, items were further screened using the mean–standard deviation threshold method (retaining items with Mj ≥ mean–standard deviation) and the coefficient of variation threshold method (removing items with CV ≥ mean + standard deviation). Discrepant cases (e.g., high-scoring items recommended for deletion) were resolved through team discussion. For psychometric validation, item analysis was performed on the pilot survey data (*n* = 113). The analyses included tendency of dispersion, discriminant, correlation coefficient, and homogeneity tests. Exploratory factor analysis (EFA) was conducted on the pilot data using principal component analysis with varimax rotation to examine the factor structure. At this stage, the selection of an varimax rotation method was guided by the primary objective of the initial scale development, namely, to obtain a clear and parsimonious factor structure that maximized the differentiation among potential dimensions, thereby facilitating their conceptual interpretation and labeling (61, 62). Subsequently, during the confirmatory factor analysis stage, the relationships among the identified dimensions were subjected to a more rigorous empirical examination. Factors with eigenvalues greater than 1, supported by the scree test and theoretical interpretability, were retained. Confirmatory factor analysis (CFA) was performed on the formal survey sample to assess the hypothesized factor structure. The goodness-of-fit index (χ^2^, df, χ^2^/df < 3), relative fit indices (TLI, CFI > 0.90), and the absolute fit index (RMSEA < 0.08) were comprehensively evaluated to assess the overall fit of the model (63, 64). Internal consistency was examined with Cronbach’s *α* and split-half reliability, and criterion-related validity was tested using Spearman’s correlations with organizational citizenship behavior (proactiveness subscale) and turnover intention. All analyses were conducted in SPSS 24.0 and AMOS 22.0, with the significance level set at α = 0.05.

## 4 Results

### 4.1 Initial item generation for the QQBS

The initial pool of items was developed through a grounded theory analysis of 34 in-depth interviews. The inter-coder reliability coefficient between the two coders was 0.86, indicating substantial agreement. The characteristics of the respondents are shown in Supplementary file 3. Following the three-level coding procedure (open, axial, and selective coding), the analysis identified two core dimensions underlying QQB—role contraction and behavioral inertia and cognitive collapse and psychological detachment (for details of the coding process and results, see Supplementary file 4). Based on the above results, an initial set of items was developed in accordance with principles of clarity, neutrality, and cultural relevance. These draft items were subsequently cross-checked against the existing literature and refined through discussions within the research team to enhance face validity and eliminate ambiguities. This process resulted in an initial pool of 25 items spanning two dimensions (see Supplementary file 5).

### 4.2 Item revision and content validity assessment of the QQBS

#### 4.2.1 Findings from the first round of Delphi expert consultation

A total of 20 questionnaires were distributed in the first round of consultation, of which 19 valid responses were received, yielding an effective response rate of 95.00%, indicating that the experts were highly enthusiastic. Supplementary file 6 presents the experts from diverse fields who participated in the consultation. The results indicated that the expert authority coefficient (Cr) was 0.90, demonstrating the high credibility of the consultation. The mean importance score (Mj) was 4.63, while the coefficient of variation (CV) ranged from 0.06 to 0.20, as shown in Supplementary file 7, reflecting strong consensus among experts. The Kendall’s coefficient of concordance (W) was 0.250 (χ^2^ = 114.143, df = 24, *p* < 0.001), further confirming the consistency of expert judgments. According to the established item screening criteria presented in Table 1, items B1, B2, B10, and B23 were excluded. Systematic revisions were made to the initial item pool based on qualitative feedback from experts and insights obtained through focus group discussions. Specifically, four items (B1, B2, B10, and B23) were removed, and six items (B3, B5, B6, B8, B14, and B18) were semantically refined to improve clarity and precision. Meanwhile, item B18 was originally classified under the dimension of cognitive collapse and psychological detachment. Following semantic refinement, it was reassigned to the dimension of role contraction and behavioral inertia. In addition, items B9 and B12, which were semantically similar, were merged into a new statement: “I believe that organizational awards and recognition have nothing to do with me.” Furthermore, two new items were added based on expert suggestions: “Even when colleagues make mistakes, I am reluctant to point them out” and “I do not care about my organization’s performance ranking.” After these revisions, the first round of consultation optimized and streamlined the scale, reducing the number of items from 25 to 22. During this process, the item codes and their sequencing were adjusted accordingly. A detailed comparison between the original and current item codes is presented in Supplementary file 8.

**Table 1:** Screening criteria for items across two rounds of expert consultation.

Expert consultation	Screening index	Mean	SD	Cut-off value
First round	Mj	4.63	0.25	<4.38
	CV	0.11	0.04	>0.15
	Full mark rate (%)	-	-	<50.00
Second round	Mj	4.82	0.26	<4.56
	CV	0.06	0.03	>0.09
	Full mark rate (%)	-	-	<50.00

#### 4.2.2 Findings from the second round of Delphi expert consultation

The second Delphi round was completed by 17 of the 19 original experts (effective response rate = 89.47%). The authority of the experts remained high (Cr = 0.96). Compared with the first round, the mean importance score (Mj) increased to 4.82, the coefficient of variation (CV) narrowed to 0.00–0.13, and Kendall’s W (W) rose markedly to 0.403 (χ^2^ = 143.894, df = 21, *p* < 0.001), indicating a high level of agreement. Detailed results are provided in Supplementary file 8. Based on the item screening criteria in Table 1, items C8 and C10 were excluded. Following focus group discussions and experts’ qualitative feedback, entries C8 and C10 were deleted, the content of C8 was integrated into C20, and this item was revised to: “I do not care about the unit’s commendations, award evaluations, or performance rankings.” After two rounds of consultation, expert opinions on the scale items had become highly consistent. The total number of scale items was ultimately reduced from 22 to 20.

### 4.3 Pilot testing and scale refinement of the QQBS

#### 4.3.1 Item analysis

A total of 113 primary healthcare workers were included in the pilot survey. The participants’ characteristics are summarized in Supplementary file 9 and are broadly consistent with the population structure of primary medical workers reported in the most recent China Health Statistical Yearbook (2023) issued by the National Health Commission of the People’s Republic of China (65). First, the dispersion analysis revealed that item C2 had a standard deviation below 0.75, indicating insufficient variability and warranting its deletion (66). Next, a discrimination analysis using independent-samples t-tests based on extreme groups (top and bottom 27%) showed that items C3-C7, C9 and C11-C22 significantly differentiated between high and low scorers (*p* < 0.001) supporting their discriminatory validity. In contrast, items C1 and C2 failed to demonstrate adequate discrimination (*p* > 0.05) and were recommended for removal (67). Furthermore, correlation analysis indicated that items with item–total correlations below 0.40 or nonsignificant relationships should be excluded (68), which led to the deletion of items C1, C2, and C19. Finally, the homogeneity test, which required that Cronbach’s *α* not increase upon item deletion (67)and that communalities exceed 0.2 with factor loadings above 0.5, also identified C1, C2, and C19 as inconsistent with the scale’s internal structure. Collectively, the convergent results from these analyses support the removal of items C1, C2, and C19, thereby enhancing the discriminant validity and internal consistency of the refined scale. The detailed results of the above project analysis are provided in Supplementary files 10–13.

#### 4.3.2 Exploratory factor analysis

An exploratory factor analysis (EFA) was performed on the 17-item QQBS using data from 113 respondents. Principal component analysis with varimax rotation was employed. The results indicated excellent suitability for factor analysis, with a KMO value of 0.922 and Bartlett’s test of sphericity reaching significance (χ^2^ = 1305.757, df = 136, *p* < 0.001). Two factors with eigenvalues greater than 1 (8.71 and 1.97, respectively) were identified and further supported by the scree plot. Items were evaluated according to the following criteria: factor loadings below 0.50, inclusion in a common factor with fewer than two items, inconsistency with the factor structure, or cross-loadings with similar values across multiple factors (69). Through iterative elimination and re-analysis, items C13 and C14 were sequentially removed. The final refined scale comprised 15 items across two factors: Factor 1 (8 items), labeled “Role Contraction and Behavioral Inertia” (explicit behavioral alienation), and Factor 2 (7 items), labeled “Cognitive Collapse and Psychological Detachment” (implicit cognitive dissonance). These two factors had eigenvalues of 7.51 and 1.97, respectively, and together explained 63.15% of the total variance, indicating a robust and interpretable factor structure. Detailed results are presented in Table 2.

**Table 2:** Exploratory factor analysis of the QQBS (*n* = 113).

Dimension	Item code	Factor 1	Factor 2	Communality
Role contraction and behavioral inertia (8 items)	C3	0.75	–	0.59
	C4	0.78	–	0.60
	C5	0.85	–	0.76
	C6	0.71	–	0.56
	C7	0.60	–	0.42
	C9	0.71	–	0.70
	C11	0.67	–	0.60
	C12	0.57	–	0.53
Cognitive collapse and psychological detachment (7 items)	C15	–	0.62	0.62
	C16	–	0.75	0.58
	C17	–	0.85	0.75
	C18	–	0.72	0.55
	C20	–	0.79	0.71
	C21	–	0.84	0.77
	C22	–	0.83	0.75
Eigenvalue		7.51	1.97	–
Variance explained (%)		32.89	30.27	–
Cumulative variance explained (%)		32.89	63.15	–

### 4.4 Formal survey and psychometric validation of the QQBS

#### 4.4.1 Confirmatory factor analysis

A total of 407 primary healthcare workers participated in the second-phase survey. The participants’ characteristics are presented in Supplementary file 14 and closely align with the population structure of primary medical workers reported in the latest China Health Statistical Yearbook (2023) released by the National Health Commission of the People’s Republic of China (65). The study constructed and compared three models based on competition theory: (M1) the single-factor model, (M2) the first-order two-factor model, and (M3) the second-order single-factor model. The results (Table 3) indicate that the first-order two-factor model (M2) provides a superior fit compared with the alternatives. Its fit indices are χ^2^/df = 2.719, Root Mean Square Error of Approximation (RMSEA) = 0.065, Root Mean Square Residual (RMR) = 0.051, Incremental Fit Index (IFI) = 0.960, Comparative Fit Index (CFI) = 0.959, Goodness of Fit Index (GFI) = 0.932, and Normed Fit Index (NFI) = 0.938, all of which meet the conventional criteria for good model fit. The first-order two-factor model (M2) was selected because it demonstrated slightly superior fit indices and offered a more parsimonious and directly interpretable representation of the construct, with its two factors clearly corresponding to the dual core manifestations of QQB.

**Table 3:** Confirmatory factor analysis of the QQBS (*n* = 407).

Model	χ^2^	df	χ^2^/df	RMSEA	RMR	IFI	CFI	GFI	NFI
M1	218.266	76	2.872	0.068	0.057	0.959	0.959	0.931	0.938
M2	220.217	81	2.719	0.065	0.051	0.960	0.959	0.932	0.938
M3	235.53	82	2.872	0.068	0.052	0.956	0.955	0.927	0.933

M1, First-order single-factor model; M2, First-order two-factor model; M3, Second-order single-factor model.

#### 4.4.2 Internal consistency

The internal consistency of the finalized QQBS was evaluated using Cronbach’s alpha and split-half reliability coefficients. The full scale demonstrated excellent reliability, with a Cronbach’s alpha of 0.921. Both subscales also showed high internal consistency, yielding alpha coefficients of 0.874 and 0.900, all exceeding the recommended threshold of 0.70 (70). To further validate these findings, the parity split-half method was employed, with corrections applied using the Spearman–Brown formula. The split-half reliability coefficient for the overall scale was 0.803, while the coefficients for the two dimensions were 0.784 and 0.848, respectively. Taken together, the results from both analytical approaches consistently indicate that the developed QQBS demonstrates high internal consistency and can be considered a stable and reliable measurement tool.

#### 4.4.3 Validity test

This study conducted a comprehensive assessment of the validity of the QQBS using multiple sources of evidence. Content validity was established through a rigorous process that included qualitative interviews with healthcare workers and two successive rounds of expert review. Structural validity was examined through a first-order two-factor confirmatory factor analysis. All standardized factor loadings ranged from 0.53 to 0.84 (Table 4), exceeding the conventional threshold of 0.40 (71). In addition, the scale demonstrates strong convergent validity. The composite reliability (CR) values for the two factors (0.866 and 0.898) demonstrate strong reliability (72). The average variance extracted (AVE) for the two factors was 0.451 and 0.560, respectively, both exceeding the threshold of 0.45 (72). For discriminant validity, the square roots of the AVEs (0.672 and 0.710) were greater than the inter-factor correlation coefficient (0.671), meeting the Fornell–Larcker criterion (73, 74). The two factors exhibited a strong correlation, consistent with their theorized linkage within the overarching construct. This correlation was lower than the square root of the AVE for each dimension, thereby satisfying the criterion for discriminant validity and supporting the distinctiveness of the two dimensions. Most importantly, the QQBS demonstrated significant criterion-related validity in the anticipated directions. The QQBS total score was significantly negatively correlated with organizational citizenship behavior (*r* = −0.561, *p* < 0.001) and significantly positively correlated with turnover intention (*r* = 0.380, *p* < 0.001), consistent with existing literature (30, 75). Collectively, these findings provide robust evidence for the validity of the QQBS. The complete list of finalized scale items and their corresponding codes is presented in Table 5.

**Table 4:** Standardized factor loadings, composite reliability (CR), and average variance extracted (AVE).

Construct	Item code	Standardized load coefficient	CR	AVE	Square root of AVE
Role contraction and behavioral inertia	C3	0.60	0.866	0.451	0.672
	C4	0.53			
	C5	0.69			
	C6	0.72			
	C7	0.56			
	C9	0.76			
	C11	0.79			
	C12	0.68			
Cognitive collapse and psychological detachment	C15	0.66	0.898	0.560	0.748
	C16	0.74			
	C17	0.76			
	C18	0.62			
	C20	0.84			
	C21	0.76			
	C22	0.83			

**Table 5:** The 15-item quiet quitting behavior scale (QQBS).

Dimension	Item code	Item description
Role contraction and behavioral inertia	C3	I do not take initiative to participate in additional work tasks.
	C4	I lower the quality and standards of my work.
	C5	I only complete tasks explicitly assigned by my supervisor.
	C6	I avoid sharing professional knowledge and experience with colleagues.
	C7	I try to minimize unnecessary work-related interactions with colleagues.
	C9	I approach work in a perfunctory manner.
	C11	I lack efficiency in my daily work.
	C12	I execute work tasks mechanically.
Cognitive collapse and psychological detachment	C15	I lack a sense of dedication in my work.
	C16	I believe the institution’s honor is irrelevant to my personal development.
	C17	I lack a sense of responsibility at work.
	C18	I lack innovative thinking in my work.
	C20	I do not care about the unit’s commendations, award evaluations, or performance rankings.
	C21	I lack a sense of identification with my work.
	C22	I lack motivation for career development at work.

## 5 Discussion

This study developed an assessment scale for QQB in the context of Chinese organizational culture and verified it among primary healthcare workers. The primary contribution of this research is the development of a dual-dimensional framework—Role Contraction and Behavioral Inertia and Cognitive Collapse and Psychological Detachment—that reinterprets quiet quitting not as passive neglect but as a deliberate strategy of adaptive resistance and self-preservation. Inductively derived from the lived experiences of Chinese employees and rigorously validated, the QQBS addresses a critical methodological gap by providing a measure that combines cultural adaptability with behavioral observability. This gap is emphasized by the conceptual distinctiveness of QQB itself (see Supplementary file 1). Unlike turnover intention or the broader notions of ‘lying flat’ or ‘slacking off,’ QQB is characterized by employees remaining in their positions while deliberately contracting their roles and psychologically disengaging. Existing scales assessing job engagement, organizational commitment, or turnover intention fail to capture this specific pattern of behavior, which persists over time and encompasses both explicit role contraction and implicit psychological withdrawal. Consequently, the QQBS provides scholars and practitioners with a reliable and valid instrument for the initial identification and assessment of this phenomenon within the context of Chinese primary healthcare.

The QQBS was developed through a rigorous multi-phase process and resulted in a 15-item scale encompassing two distinct dimensions. The first dimension, role contraction and behavioral inertia, includes eight items (C3, C4, C5, C6, C7, C9, C11, and C12). The second dimension, cognitive collapse and psychological detachment, consists of seven items (C15, C16, C17, C18, C20, C21, and C22). As a self-report measurement instrument, the scale adopts a five-point Likert response format, ranging from 1 to 5 (strongly disagree-strongly agree) and contains no reverse-scored items. Higher overall mean scores indicate greater severity of employees’ QQB. In addition, scores for each subdimension can be calculated separately according to the dimensional structure of the scale. Comprehensive psychometric analyses confirmed the scale’s strong reliability and validity, including satisfactory content validity, a stable two-factor structure, high internal consistency, and expected correlations with related constructs. Although the average variance extracted (AVE = 0.451) for the first dimension is slightly below the recommended threshold of 0.50, it meets the more lenient criterion of 0.45 adopted in comparable studies (72) and demonstrates strong composite reliability (CR = 0.866). From a methodological perspective, the CR value for this dimension is high (0.866), indicating strong internal consistency and providing robust evidence of convergent validity. Prior research suggests that when CR reaches an adequate level, convergent validity can still be regarded as acceptable even if AVE falls slightly below the conventional threshold of 0.50 (76–78). From a theoretical perspective, the slightly lower average variance extracted (AVE) for this dimension may stem from the nature of the construct itself. Role contraction and behavioral inertia constitute a spectrum of coping strategies that range from active refusal to passive avoidance. The heterogeneity of behavioral manifestations captured by this dimension may lead to a modest reduction in the proportion of variance explained by a single latent factor, as reflected in the average variance explained (AVE). Future research may refine the items within this dimension to further improve convergent validity. Collectively, these findings support the QQBS as a reliable and valid tool for evaluating QQB among primary healthcare workers in China.

A key conceptual strength of this scale lies in its explicit operationalization of QQB at the behavioral level, rather than reducing it to psychological states or abstract processes. This approach aligns with the construct’s theoretical definition as a conscious, strategic effort to renegotiate responsibility boundaries. Each item captures concrete, observable actions or deliberate cognitive refusals, thereby enhancing the scale’s capacity for objective measurement and practical applicability. Meanwhile, the two-dimensional structure offers not only empirical soundness but also theoretical richness, enabling a nuanced understanding of QQB. Specifically, Cognitive Dissonance and Labor Alienation theories account for the motivational basis of role contraction and behavioral inertia, whereas Implicit Cognition and Suspension theories explain the automatic and sustained nature of cognitive collapse and psychological detachment. By integrating the external behavioral strategy of role contraction with the internal cognitive–affective process of detachment, the two-dimensional model reconceptualizes QQB not as passive neglect but as a deliberate strategy of adaptive resistance and self-preservation. This comprehensive perspective provides researchers and practitioners with a nuanced framework for accurately identifying and effectively addressing the phenomenon within organizational contexts.

The QQBS demonstrates considerable practical value for organizational management and human resource practices. As highlighted in a recent Lancet series, the workplace serves as a critical environment for protecting employee well-being (79–81), and QQB poses a significant threat to achieving this objective. Within this framework, the QQBS provides managers and HR professionals in primary healthcare with a precise diagnostic and early-warning instrument. It enables hospital administrators to systematically monitor and address the increasing prevalence of QQ among healthcare professionals (1, 11, 13), thereby contributing to the mitigation of burnout and the maintenance of workforce stability in this essential sector. In practice, the QQBS can be integrated into routine employee engagement surveys to identify teams with elevated risk profiles, facilitating timely and targeted managerial interventions. Its dual-factor structure further offers nuanced guidance for targeted responses. For instance, issues within the Role Contraction and Behavioral Inertia dimension may require initiatives to clarify roles and enhance leader–member exchange, whereas challenges in the Cognitive Collapse and Psychological Detachment dimension may necessitate efforts to restore psychological meaningfulness and rebuild trust.

The QQBS provides distinct advantages over existing measures of quiet quitting, particularly in its theoretical grounding, cultural adaptability, and behavioral observability. First, its theoretical foundation is comprehensive. Unlike scales that typically rely on a single perspective, the QQBS is supported by an integrated framework of four foundational theories. Cognitive Dissonance Theory and Labor Alienation explain the behavioral strategy of role contraction, while Implicit Cognition Theory and Suspended Theory elucidate the psychological state of detachment. This multifaceted foundation enables a more holistic understanding of the phenomenon than unidimensional interpretations. Second, the QQBS demonstrates superior cultural adaptability. Existing instruments, often developed within individualistic paradigms (e.g., Anand et al.’s (26) scale focusing on psychological commitment) or other culture contexts (28, 33), may fail to capture culturally nuanced behaviors. By contrast, the QQBS is inductively derived from the lived experiences of Chinese employees and refined through input from local experts. It is thus uniquely capable of assessing culture-specific, non-confrontational manifestations of quiet quitting—such as superficial compliance coupled with strategic delay—characteristic of collectivist workplaces. Finally, the QQBS ensures rigorous behavioral observability. Consistent with its conceptual definition, all items describe concrete, observable actions or directly inferable stances. This avoids a common shortcoming of existing tools, which often conflate behaviors with their antecedents [e.g., lack of motivation (28)] or outcomes [e.g., turnover intention scales (7)]. Overall, the QQBS provides a theoretically robust, culturally contextualized, and behaviorally precise measure.

This study provides a validated instrument but is not without limitations, which also suggest directions for future research. First, the generalizability of the results may be limited by the sample. Although the target population was theoretically significant, the validation was conducted exclusively among primary health care workers in China. The primary objective of future research is to conduct systematic cross-group and cross-cultural validation. Specifically, the QQBS should be applied to other high-stress occupational groups (e.g., teachers, IT professionals, and emergency service personnel), as well as to medical workers from diverse cultural contexts, in order to assess cross-sample validity and establish cross-cultural measurement equivalence. Achieving measurement equivalence requires a series of methodological procedures, including cognitive interviews, differential item functioning (DIF) analyses, and multi-group CFA. Second, although this study implemented procedural remedies, such as ensuring respondent anonymity and randomizing item order during data collection, to mitigate common method bias, inherent limitations associated with cross-sectional, self-reported data persist. Future research could strengthen the evidence base by incorporating multi-source ratings, such as supervisor or peer assessments of QQBS. Third, and most importantly, the cross-sectional design of this survey precludes drawing definitive conclusions about causality. The associations identified between quiet quitting and other variables (e.g., organizational citizenship behavior, turnover intention) remain correlational; longitudinal or experimental studies are needed to disentangle causal mechanisms and establish the temporal ordering of antecedents. Meanwhile, future research should examine the validity of the QQBS within a broader criterion network. Specifically, incorporating a wider range of work attitudes and behaviors—such as job burnout, psychological contract breach, work engagement, job satisfaction, organizational commitment, and performance indicators—would enable a more comprehensive understanding of the antecedents and consequences of QQB. In addition, future studies should develop evidence-based guidelines for the practical application of the QQBS, including the identification of optimal screening parameters, the establishment of clinically meaningful risk thresholds, and the evaluation of the effectiveness of profile-informed interventions.

## 6 Conclusion

This study successfully developed the QQBS and validated it among primary healthcare workers in China, providing a reliable and culturally appropriate measurement scale. Its two-dimensional structure offers a comprehensive framework that integrates both behavioral and psychological aspects of the phenomenon, providing deeper insights than unidimensional alternatives. By providing a reliable instrument to assess this universal issue, QQBS establishes a solid foundation for future academic research and effective organizational practices aimed at improving the mental and behavioral health of the workforce and fostering a sustainable working environment, particularly in high-stress sectors such as healthcare.

## Data Availability

The original contributions presented in the study are included in the article/Supplementary material, further inquiries can be directed to the corresponding author.
