# Supplementary material for: Development and validation of the quiet quitting behavior scale: a mixed-methods study with primary healthcare workers in China
Source: Front Public Health. 2026 Mar 12;14:1773183. doi: 10.3389/fpubh.2026.1773183 (PMC13017915; doi:10.3389/fpubh.2026.1773183)
Supplement: Supplementary file 1 [file Table_1.DOCX]

**Supplementary File 1 Conceptual Differentiation of QQB**

| **Dimension** | **QQB** | **Lying Flat** | **Slacking Off** | **Turnover Behavior** | **Turnover Intention** |
| --- | --- | --- | --- | --- | --- |
| **Core Feature** | Psychological withdrawal with physical presence | Systematic rejection of social competition norms | Situational adjustment of work intensity | Physical termination of organizational ties | Psychological predisposition toward departure |
| **Specific Manifestation** | Minimal compliance; avoidance of extra effort | Low-desire lifestyle choices | Intermittent passive task execution | Physical disengagement from position | Inclination toward psychological exit |
| **Underlying Mechanism** | Instrumental rationality–driven adaptive strategy | Broad retreat amid diminished value rationality | Cognitive regulation of workload | Decisive commitment to employment termination | Cognitive processing of dissatisfaction |
| **Evolutionary Trait** | Gradual disengagement | Systematic withdrawal | Contextual adjustment | Abrupt detachment | Accumulated threshold breach |
| **Organizational Impact** | Implicit reduction of service effectiveness | Explicit conflict with organizational culture | Localized fluctuations in work efficiency | Direct loss of human capital | Potential risk of talent attrition |
