# Supplementary material for: Development and validation of the quiet quitting behavior scale: a mixed-methods study with primary healthcare workers in China
Source: Front Public Health. 2026 Mar 12;14:1773183. doi: 10.3389/fpubh.2026.1773183 (PMC13017915; doi:10.3389/fpubh.2026.1773183)
Supplement: Supplementary file 2 [file Table_2.DOCX]

**Supplementary File 2 Interview Guide for the Qualitative Phase**

**Introduction and Background Information**

This section introduces the research topic and objectives and collects essential background information about the respondents.

1. Provide a brief explanation of the research topic and purpose, and confirm informed consent.

2. Collect basic background information about the respondents (e.g., position, role, and length of service).

3. Obtain a brief description of the institution and the respondents’ working environment.

**Core Interview Sections**

**I. Work Experience and Responsibility Boundaries (Exploratory Entry Point)**

This section seeks to understand respondents’ overall work context and their perceptions of job responsibilities.

1. Could you describe the main components of your daily work? What is your overall impression of this role?

2. How do you define the scope of your job responsibilities? Which tasks do you consider part of your formal duties, and which do you perceive as requiring additional effort beyond your role?

**II. General Observations on Work Engagement and Behavioral Changes (Natural Transition)**

This section explores changes in work engagement, as perceived by respondents themselves and as observed among their colleagues.

1. Compared with the past, have you noticed any changes in your willingness or behavior regarding the investment of time and energy to achieve high work performance? Please describe the nature of these changes.

2. Have you observed any changes in your colleagues’ work commitment, enthusiasm, or sense of responsibility? Please elaborate.

**III. Introduction and Interpretation of the Concept of Quiet Quitting Behavior (Core Construct Exploration)**

This section introduces the focal concept and explores localized interpretations, aligning with the core analytical framework of the study.

1. Have you heard of the concept of Quiet Quitting Behavior? How do you understand it?

2. Based on your understanding, do you think Quiet Quitting Behavior is present in your own work practices or among your colleagues? Please provide an illustrative example.

**IV. Personal Strategies and Specific Examples**

Building on the shared understanding established in the previous section, this section examines specific behaviors and decision-making processes when respondents encounter tasks that exceed clearly defined responsibilities.

1. When you are assigned or asked to complete tasks that fall outside your formal responsibilities, how do you typically assess and respond to such situations? Please provide specific examples.

2. Do you ever take the initiative to assume additional responsibilities or voluntarily assist with team- or organization-related tasks? What factors influence your decision to do so?

**V. Organizational Interaction and Cognitive Changes**

This section examines respondents’ perceptions of organizational systems, particularly evaluation and performance appraisal mechanisms.

1. How do you perceive and engage with practices such as performance rankings or awards within your organization? To what extent, if any, do these practices influence your attitudes, motivation, or behavior at work?

2. Over recent years, have you experienced any changes in how you perceive the meaning or value of your work? If so, please describe the nature of these changes.

**VI. Additional Comments**

1. Is there anything else you would like to add regarding your work experiences or the changes we discussed today?
