# Supplementary material for: Development and validation of the quiet quitting behavior scale: a mixed-methods study with primary healthcare workers in China
Source: Front Public Health. 2026 Mar 12;14:1773183. doi: 10.3389/fpubh.2026.1773183 (PMC13017915; doi:10.3389/fpubh.2026.1773183)
Supplement: Supplementary file 3 [file Table_3.DOCX]

**Supplementary File 3 Demographic Information of Interviewees (n=34)**

| **Variable** | **Category** | **n** | **%** |
| --- | --- | --- | --- |
| Sex | Male | 22 | 64.71 |
|  | Female | 12 | 35.29 |
| Age | ≤25 | 2 | 5.88 |
|  | 26–35 | 8 | 23.53 |
|  | 36–45 | 13 | 38.24 |
|  | 46–55 | 11 | 32.35 |
|  | >55 | 0 | 0.00 |
| Years of Work | <6 | 5 | 14.71 |
|  | 6-10 | 4 | 11.76 |
|  | 11-15 | 8 | 23.53 |
|  | 16-20 | 2 | 5.88 |
|  | >20 | 15 | 44.12 |
| Education Level | Junior high school or below | 0 | 0 |
|  | High School or Secondary School | 6 | 17.65 |
|  | Associate Degree | 17 | 50.00 |
|  | Bachelor’s Degree | 10 | 29.41 |
|  | Master’s or Above | 1 | 2.94 |
| Professional Title | No Title | 1 | 2.94 |
|  | Junior | 4 | 11.76 |
|  | Intermediate | 23 | 67.65 |
|  | Senior | 6 | 17.65 |
| Industry | Healthcare | 6 | 17.65 |
|  | Education | 5 | 14.71 |
|  | Technology | 6 | 17.65 |
|  | Manufacturing | 7 | 20.59 |
|  | Public services | 10 | 29.41 |
| Total |  | 34 | 100.00 |
