# Supplementary material for: Development and validation of the quiet quitting behavior scale: a mixed-methods study with primary healthcare workers in China
Source: Front Public Health. 2026 Mar 12;14:1773183. doi: 10.3389/fpubh.2026.1773183 (PMC13017915; doi:10.3389/fpubh.2026.1773183)
Supplement: Supplementary file 4 [file Table_4.DOCX]

**Supplementary File 4 Coding process**

| **Open coding** | **Axial coding** | **Selective coding** |
| --- | --- | --- |
| a1 Knowledge retention, a2 Social isolation, a3 Experiential isolation, a4 Information isolation | A1 Avoidance of knowledge sharing and interaction | Role contraction and behavioral inertia |
| a5 Role fixation, a6 Task limitation, a7 Refusal of additional responsibilities, a8 Shirking extra tasks, a9 Excessive sense of responsibility boundaries, a10 Low work participation, a11 Disregard for unassigned tasks, a12 Indifference toward tasks beyond one’s scope | A2 Unwillingness to exceed job scope |  |
| a13 Work resistance, a14 Psychological unpreparedness, a15 Work avoidance | A3 Unwillingness to confront work |  |
| a16 Passive task execution, a17 Lack of initiative, a18 Avoidance of additional work | A4 Lack of proactivity in extra work |  |
| a19 Lowered work standards, a20 Reduced work quality, a21 Decreased work input, a22 Relaxed work requirements | A5 Lowering of work standards |  |
| a23 Mechanical compliance, a24 Mere adherence, a25 Lack of motivation | A6 Passive compliance with work |  |
| a26 Indifference toward unrelated matters, a27 Apathy toward performance, a28 Detachment from work | A7 Indifferent attitude toward work |  |
| a29 Superficial effort, a30 Lack of initiative | A8 Perfunctory work performance |  |
| a31 Lack of willingness for personal growth, a32 Absence of entrepreneurial spirit, a33 Contentment with current position | A9 Settling for the current work situation |  |
| a34 Difficulty adapting to technology, a35 Lack of learning motivation, a36 Decline in learning enthusiasm, a37 Low willingness to learn, a38 Slow knowledge acquisition, a39 Insufficient learning capacity | A10 Lack of learning ability |  |
| a40 Task procrastination, a41 Passive attitude, a42 Weak execution ability | A11 Lack of execution ability |  |
| a43 Low participation in activities, a44 Prioritization of personal interests | A12 Lack of dedication | Cognitive collapse and psychological detachment |
| a45 Innovation stagnation, a46 Maintenance of status quo | A13 Lack of innovativeness |  |
| a47 Weak collective consciousness, a48 Neglect of group responsibility, a49 Absence of collective honor | A14 Lack of collective honor |  |
| a50 Diminished enthusiasm, a51 Deviation in beliefs, a52 Fluctuating commitment | A15 Lack of enthusiasm for work |  |
| a53 Weak identification with work, a54 Unrealized self-worth | A16 Lack of work identity |  |
| a55 Insufficient responsibility, a56 Carelessness, a57 Neglect of quality, a58 Irresponsibility toward patients | A17 Lack of responsibility |  |
| a59 Low motivation for personal development, a60 Unclear career goals | A18 Lack of promotion motivation |  |
| a61 Psychological withdrawal, a62 Physical presence without engagement, a63 Poor concentration | A19 Present in body but absent in mind |  |
