# Supplementary material for: Development and validation of the quiet quitting behavior scale: a mixed-methods study with primary healthcare workers in China
Source: Front Public Health. 2026 Mar 12;14:1773183. doi: 10.3389/fpubh.2026.1773183 (PMC13017915; doi:10.3389/fpubh.2026.1773183)
Supplement: Supplementary file 5 [file Table_5.docx]

**Supplementary File 5 The initial pool of items for the quiet quitting behavior**

| **Dimension** | **Item Description** | **Item Source** |
| --- | --- | --- |
| Role Contraction and Behavioral Inertia | B1 I have found effective ways to manage stress and maintain a positive attitude at work. | Anand A [1], Focus Group Discussions |
|  | B2 I actively seek ways to balance work and personal life. | Supplemented from Focus Group Discussions |
|  | B3 I deliberately avoid sharing professional knowledge and experience with colleagues. | Category A1 |
|  | B4 I try to minimize unnecessary work-related interactions with colleagues. | Category A1 |
|  | B5 I only fulfill the minimum job requirements and avoid taking on additional tasks. | Category A2, Anand A [1] |
|  | B6 I am unwilling to face challenges and pressures at work. | Category A3 |
|  | B7 I do not take initiative to participate in additional work tasks. | Category A4, Galanis P [2] |
|  | B8 I lower my work quality standards to conserve energy. | Category A5 |
|  | B9 I adopt a perfunctory attitude and do not strive for improvement. | Category A6 |
|  | B10 I am indifferent to work outcomes and team interests. | Category A7 |
|  | B11 I approach with work in a perfunctory manner. | Category A8 |
|  | B12 I lack initiative and ambition in my work. | Category A9 |
|  | B13 I lack motivation to learn new knowledge and skills. | Category A10 |
|  | B14 I lack efficiency and determination in performing tasks. | Category A11 |
| Cognitive Collapse and Psychological Detachment | B15 I lack a sense of dedication in my work. | Category A12 |
|  | B16 I lack innovation thinking in my work. | Category A13 |
|  | B17 I believe the institution’s honor is irrelevant to my personal development. | Category A14 |
|  | B18 I feel passionate about my work. (reverse-coded) | Category A15, Anand A [1] |
|  | B19 I lack a sense of identification with my work. | Category A16 |
|  | B20 I lack a sense of responsibility at work. | Category A17 |
|  | B21 I lack motivation for career development at work. | Category A18 |
|  | B22 Although I am present at work, my mind is not focused on it. | Category A19 |
|  | B23 I find it difficult to concentrate on work. | Anand A [1], Focus Group Discussions |
|  | B24 I feel emotionally detached from my work. | Supplemented from Focus Group Discussions |
|  | B25 I no longer feel joy or disappointment about my work results. | Supplemented from Focus Group Discussions |

**Reference**

[1] Anand A, Doll J, Ray P: **Drowning in silence: a scale development and validation of quiet quitting and quiet firing**. *International Journal of Organizational Analysis* 2024, **32**(4): 721-43.

[2] Galanis P, Katsiroumpa A, Vraka, I, Siskou O, Konstantakopoulou O, & Moisoglou I, Gallos P, Kaitelidou D: **The quiet quitting scale: Development and initial validation.** *AIMS public health* 2023, **10**(4): 828-48.
