# Supplementary material for: Development and validation of the quiet quitting behavior scale: a mixed-methods study with primary healthcare workers in China
Source: Front Public Health. 2026 Mar 12;14:1773183. doi: 10.3389/fpubh.2026.1773183 (PMC13017915; doi:10.3389/fpubh.2026.1773183)
Supplement: Supplementary file 6 [file Table_6.DOCX]

**Supplementary File 6 Basic Information of Experts in the Delphi Consultation (N=19)**

| **Variable** | **Category** | **n** | **%** |
| --- | --- | --- | --- |
| Sex | Male | 15 | 78.95 |
|  | Female | 4 | 21.05 |
| Age | 31-40 | 4 | 21.05 |
|  | 41-50 | 10 | 52.63 |
|  | 51-60 | 5 | 26.32 |
| Years of Work | 6-10 | 2 | 10.53 |
|  | 11-20 | 10 | 52.63 |
|  | 21-30 | 5 | 26.32 |
|  | Over 30 | 2 | 10.53 |
| Professional Title | Senior Title | 12 | 63.16 |
|  | Associate Senior Title | 7 | 36.84 |
| Education Level | Master’s Degree | 3 | 15.79 |
|  | Doctoral Degree | 16 | 84.21 |
| Field of Expertise | Management | 9 | 47.37 |
|  | psychology | 7 | 36.84 |
|  | Organizational Behavior | 3 | 15.79 |
| **Total** |  | **19** | **100.00** |
