# Supplementary material for: Development and validation of the quiet quitting behavior scale: a mixed-methods study with primary healthcare workers in China
Source: Front Public Health. 2026 Mar 12;14:1773183. doi: 10.3389/fpubh.2026.1773183 (PMC13017915; doi:10.3389/fpubh.2026.1773183)
Supplement: Supplementary file 7 [file Table_7.docx]

**Supplementary File 7 Item scores from the first round of Delphi expert consultation**

| Dimension | Item Code | Item Description | Mean | SD | CV | Full Score Rate (%) |
| --- | --- | --- | --- | --- | --- | --- |
| Role Contraction and Behavioral Inertia | B1 | I have found effective ways to manage stress and maintain a positive attitude at work. | 4.00 | 0.67 | 0.17 | 78.95 |
|  | B2 | I actively seek ways to balance work and personal life. | 3.95 | 0.78 | 0.20 | 68.42 |
|  | B3 | I deliberately avoid sharing professional knowledge and experience with colleagues. | 4.89 | 0.32 | 0.06 | 100.00 |
|  | B4 | I try to minimize unnecessary work-related interactions with colleagues. | 4.89 | 0.32 | 0.06 | 100.00 |
|  | B5 | I only fulfill the minimum job requirements and avoid taking on additional tasks. | 4.79 | 0.54 | 0.11 | 94.74 |
|  | B6 | I am unwilling to face challenges and pressures at work. | 4.84 | 0.38 | 0.08 | 100.00 |
|  | B7 | I do not take initiative to participate in additional work tasks. | 4.74 | 0.45 | 0.10 | 100.00 |
|  | B8 | I lower my work quality standards to conserve energy. | 4.74 | 0.45 | 0.10 | 100.00 |
|  | B9 | I adopt a perfunctory attitude and do not strive for improvement. | 4.84 | 0.38 | 0.08 | 100.00 |
|  | B10 | I am indifferent to work outcomes and team interests. | 4.42 | 0.69 | 0.16 | 89.47 |
|  | B11 | I approach work in a perfunctory manner. | 4.89 | 0.32 | 0.06 | 100.00 |
|  | B12 | I lack initiative and ambition in my work. | 4.79 | 0.54 | 0.11 | 94.74 |
|  | B13 | I lack motivation to learn new knowledge and skills. | 4.63 | 0.60 | 0.13 | 94.74 |
|  | B14 | I lack efficiency and determination in performing tasks. | 4.74 | 0.45 | 0.10 | 100.00 |
| Cognitive Collapse and Psychological Detachment | B15 | I lack a sense of dedication in my work. | 4.53 | 0.70 | 0.15 | 89.47 |
|  | B16 | I lack innovation thinking in my work. | 4.58 | 0.51 | 0.11 | 100.00 |
|  | B17 | I believe the institution’s honor is irrelevant to my personal development. | 4.84 | 0.38 | 0.08 | 100.00 |
|  | B18 | I feel passionate about my work. (reverse-coded) | 4.58 | 0.69 | 0.15 | 89.47 |
|  | B19 | I lack a sense of identification with my work. | 4.68 | 0.58 | 0.12 | 94.74 |
|  | B20 | I lack a sense of responsibility at work. | 4.58 | 0.69 | 0.15 | 89.47 |
|  | B21 | I lack motivation for career development at work. | 4.63 | 0.60 | 0.13 | 94.74 |
|  | B22 | Although I am present at work, my mind is not focused on it. | 4.53 | 0.61 | 0.14 | 94.74 |
|  | B23 | I find it difficult to concentrate on work. | 4.37 | 0.60 | 0.14 | 94.74 |
|  | B24 | I feel emotionally detached from my work. | 4.58 | 0.51 | 0.11 | 100.00 |
|  | B25 | I no longer feel joy or disappointment about my work results. | 4.79 | 0.42 | 0.09 | 100.00 |
