# Supplementary material for: Development and validation of the quiet quitting behavior scale: a mixed-methods study with primary healthcare workers in China
Source: Front Public Health. 2026 Mar 12;14:1773183. doi: 10.3389/fpubh.2026.1773183 (PMC13017915; doi:10.3389/fpubh.2026.1773183)
Supplement: Supplementary file 8 [file Table_8.docx]

**Supplementary File 8 Item scores from the second round of Delphi expert consultation**

| Dimension | Original code | Current code | Item Description | Mean | SD | CV | Full Score Rate (%) |
| --- | --- | --- | --- | --- | --- | --- | --- |
| Role Contraction and Behavioral Inertia | B6 | C1 | I choose to avoid handling unexpected incidents at work. | 5.00 | 0.00 | 0.00 | 100.00 |
|  | New addition | C2 | Even when colleagues make mistakes, I am reluctant to point them out. | 4.94 | 0.24 | 0.05 | 100.00 |
|  | B7 | C3 | I do not take initiative to participate in additional work tasks. | 4.94 | 0.24 | 0.05 | 100.00 |
|  | B8 | C4 | I lower the quality and standards of my work. | 4.76 | 0.44 | 0.09 | 100.00 |
|  | B5 | C5 | I only complete tasks explicitly assigned by my supervisor. | 5.00 | 0.00 | 0.00 | 100.00 |
|  | B3 | C6 | I avoid sharing professional knowledge and experience with colleagues. | 4.94 | 0.24 | 0.05 | 100.00 |
|  | B4 | C7 | I try to minimize unnecessary work-related interactions with colleagues. | 4.88 | 0.33 | 0.07 | 100.00 |
|  | B9, B12 | C8 | I believe that organizational awards and recognition have nothing to do with me. | 4.24 | 0.44 | 0.10 | 100.00 |
|  | B11 | C9 | I approach work in a perfunctory manner. | 4.76 | 0.44 | 0.09 | 100.00 |
|  | B13 | C10 | I lack motivation to learn new knowledge and skills. | 3.88 | 0.49 | 0.13 | 58.82 |
|  | B14 | C11 | I lack efficiency in my daily work. | 4.94 | 0.24 | 0.05 | 100.00 |
|  | B18 | C12 | I execute work tasks mechanically. | 4.94 | 0.24 | 0.05 | 100.00 |
| Cognitive Collapse and Psychological Detachment | B22 | C13 | Although I am present at work, my mind is not focused on it. | 4.94 | 0.24 | 0.05 | 100.00 |
|  | B24 | C14 | I feel emotionally detached from my work. | 4.88 | 0.33 | 0.07 | 100.00 |
|  | B15 | C15 | I lack a sense of dedication in my work. | 4.88 | 0.33 | 0.07 | 100.00 |
|  | B17 | C16 | I believe the institution’s honor is irrelevant to my personal development. | 4.88 | 0.33 | 0.07 | 100.00 |
|  | B20 | C17 | I lack a sense of responsibility at work. | 4.88 | 0.33 | 0.07 | 100.00 |
|  | B16 | C18 | I lack innovative thinking in my work. | 4.88 | 0.33 | 0.07 | 100.00 |
|  | B25 | C19 | I no longer feel joy or disappointment about my work results. | 4.94 | 0.24 | 0.05 | 100.00 |
|  | New addition | C20 | I do not care about my organization’s performance ranking. | 4.76 | 0.44 | 0.09 | 100.00 |
|  | B19 | C21 | I lack a sense of identification with my work. | 4.94 | 0.24 | 0.05 | 100.00 |
|  | B21 | C22 | I lack motivation for career development at work. | 4.94 | 0.24 | 0.05 | 100.00 |
