# Supplementary material for: Development and validation of the quiet quitting behavior scale: a mixed-methods study with primary healthcare workers in China
Source: Front Public Health. 2026 Mar 12;14:1773183. doi: 10.3389/fpubh.2026.1773183 (PMC13017915; doi:10.3389/fpubh.2026.1773183)
Supplement: Supplementary file 9 [file Table_9.DOCX]

**Supplementary File 9 Demographic characteristics of primary medical staff in the pilot survey (n=113)**

| **Variable** | **Category** | **n** | **%** |
| --- | --- | --- | --- |
| Gender | Male | 52 | 46.02 |
|  | Female | 61 | 53.98 |
| Age | ≤25 | 10 | 8.85 |
|  | 26–35 | 55 | 48.67 |
|  | 36–45 | 34 | 30.09 |
|  | 46–55 | 9 | 7.96 |
|  | >55 | 5 | 4.42 |
| Marital Status | Married | 90 | 79.65 |
|  | Unmarried | 18 | 15.93 |
|  | Divorced | 4 | 3.54 |
|  | Other | 1 | 0.88 |
| Years of Work | ≤5 | 30 | 26.55 |
|  | 6–10 | 37 | 32.74 |
|  | 11–15 | 18 | 15.93 |
|  | 16–20 | 12 | 10.62 |
|  | >20 | 16 | 14.16 |
| Education Level | Junior high school or below | 0 | 0 |
|  | High School or Secondary School | 18 | 15.93 |
|  | Associate Degree | 52 | 46.02 |
|  | Bachelor’s Degree | 41 | 36.28 |
|  | Master’s or Above | 2 | 1.77 |
| Professional Title | None | 6 | 5.31 |
|  | Junior | 35 | 30.97 |
|  | Intermediate | 52 | 46.02 |
|  | Senior | 20 | 17.70 |
| Monthly Income (RMB) | <2,000 | 2 | 1.77 |
|  | 2,000–3,000 | 40 | 35.40 |
|  | 3,001–4,000 | 41 | 36.28 |
|  | 4,001–5,000 | 21 | 18.58 |
|  | >5,000 | 9 | 7.96 |
| Total |  | 113 | 100.00 |
