# Supplementary material for: Development and validation of the quiet quitting behavior scale: a mixed-methods study with primary healthcare workers in China
Source: Front Public Health. 2026 Mar 12;14:1773183. doi: 10.3389/fpubh.2026.1773183 (PMC13017915; doi:10.3389/fpubh.2026.1773183)
Supplement: Supplementary file 10 [file Table_10.docx]

**Supplementary File 10 The mean and standard deviation of each scale item in the pilot survey (n = 113).**

| Item code | Mean | SD |
| --- | --- | --- |
| C1 | 4.25 | 0.75 |
| C2 | 4.36 | 0.66 |
| C3 | 2.44 | 1.34 |
| C4 | 2.60 | 1.29 |
| C5 | 2.16 | 1.33 |
| C6 | 1.86 | 1.13 |
| C7 | 2.54 | 1.09 |
| C9 | 2.22 | 1.27 |
| C11 | 2.49 | 1.31 |
| C12 | 2.39 | 1.21 |
| C13 | 2.14 | 1.05 |
| C14 | 2.18 | 1.14 |
| C15 | 2.18 | 1.14 |
| C16 | 1.73 | 1.07 |
| C17 | 1.62 | 0.92 |
| C18 | 2.19 | 1.13 |
| C19 | 2.35 | 1.14 |
| C20 | 1.84 | 0.97 |
| C21 | 1.94 | 1.03 |
| C22 | 1.61 | 0.94 |
