# Supplementary material for: Development and validation of the quiet quitting behavior scale: a mixed-methods study with primary healthcare workers in China
Source: Front Public Health. 2026 Mar 12;14:1773183. doi: 10.3389/fpubh.2026.1773183 (PMC13017915; doi:10.3389/fpubh.2026.1773183)
Supplement: Supplementary file 11 [file Table_11.docx]

**Supplementary File 11 Independent sample t-tests comparing score differences in the scale’s initial items between high and low groups (n = 113).**

| Item code | Group | *M* | *SD* | *CR(t)* |
| --- | --- | --- | --- | --- |
| C1 | High score group | 4.18 | 0.81 | -1.72 |
|  | Low score group | 4.50 | 0.67 |  |
| C2 | High score group | 4.24 | 0.75 | -1.56 |
|  | Low score group | 4.50 | 0.57 |  |
| C3 | High score group | 3.48 | 1.00 | 9.04*** |
|  | Low score group | 1.38 | 0.87 |  |
| C4 | High score group | 3.48 | 1.12 | 7.13*** |
|  | Low score group | 1.59 | 1.01 |  |
| C5 | High score group | 3.39 | 1.06 | 11.85*** |
|  | Low score group | 1.09 | 0.30 |  |
| C6 | High score group | 2.70 | 1.26 | 7.39*** |
|  | Low score group | 1.03 | 0.18 |  |
| C7 | High score group | 3.33 | 0.92 | 6.81*** |
|  | Low score group | 1.75 | 0.95 |  |
| C9 | High score group | 3.52 | 1.12 | 11.56*** |
|  | Low score group | 1.13 | 0.34 |  |
| C11 | High score group | 3.76 | 0.97 | 11.37*** |
|  | Low score group | 1.28 | 0.77 |  |
| C12 | High score group | 3.55 | 0.87 | 10.10*** |
|  | Low score group | 1.41 | 0.84 |  |
| C13 | High score group | 3.15 | 1.00 | 10.07*** |
|  | Low score group | 1.22 | 0.42 |  |
| C14 | High score group | 3.27 | 1.01 | 9.95*** |
|  | Low score group | 1.28 | 0.52 |  |
| C15 | High score group | 3.30 | 0.88 | 11.99*** |
|  | Low score group | 1.19 | 0.47 |  |
| C16 | High score group | 2.55 | 1.28 | 6.85*** |
|  | Low score group | 1.00 | 0.00 |  |
| C17 | High score group | 2.36 | 1.17 | 6.38*** |
|  | Low score group | 1.03 | 0.18 |  |
| C18 | High score group | 3.06 | 1.09 | 8.55*** |
|  | Low score group | 1.25 | 0.51 |  |
| C19 | High score group | 2.91 | 1.01 | 4.58*** |
|  | Low score group | 1.72 | 1.09 |  |
| C20 | High score group | 2.73 | 1.01 | 9.37*** |
|  | Low score group | 1.03 | 0.18 |  |
| C21 | High score group | 3.00 | 1.03 | 9.54*** |
|  | Low score group | 1.16 | 0.37 |  |
| C22 | High score group | 2.45 | 1.18 | 7.00*** |
|  | Low score group | 1.00 | 0.00 |  |

注：****P*＜0.001。
