# Supplementary material for: Development and validation of the quiet quitting behavior scale: a mixed-methods study with primary healthcare workers in China
Source: Front Public Health. 2026 Mar 12;14:1773183. doi: 10.3389/fpubh.2026.1773183 (PMC13017915; doi:10.3389/fpubh.2026.1773183)
Supplement: Supplementary file 12 [file Table_12.docx]

**Supplementary File 12 Correlation between Initial item scores and total scale score, and corrected item total correlations (n = 113)**

| Item code | Correlation with the total score of the scale | *P* | Total correlation of the corrected items |
| --- | --- | --- | --- |
| C1 | -0.08 | 0.418 | -0.13 |
| C2 | -0.08 | 0.392 | -0.13 |
| C3 | 0.65*** | 0.000 | 0.59 |
| C4 | 0.58*** | 0.000 | 0.51 |
| C5 | 0.72*** | 0.000 | 0.67 |
| C6 | 0.66*** | 0.000 | 0.61 |
| C7 | 0.61*** | 0.000 | 0.55 |
| C9 | 0.80*** | 0.000 | 0.76 |
| C11 | 0.76*** | 0.000 | 0.72 |
| C12 | 0.72*** | 0.000 | 0.67 |
| C13 | 0.77*** | 0.000 | 0.73 |
| C14 | 0.80*** | 0.000 | 0.77 |
| C15 | 0.77*** | 0.000 | 0.73 |
| C16 | 0.62*** | 0.000 | 0.57 |
| C17 | 0.70*** | 0.000 | 0.67 |
| C18 | 0.62*** | 0.000 | 0.56 |
| C19 | 0.45*** | 0.000 | 0.38 |
| C20 | 0.76*** | 0.000 | 0.72 |
| C21 | 0.75*** | 0.000 | 0.72 |
| C22 | 0.74*** | 0.000 | 0.71 |

注：****P*＜0.001。
