# Supplementary material for: Development and validation of the quiet quitting behavior scale: a mixed-methods study with primary healthcare workers in China
Source: Front Public Health. 2026 Mar 12;14:1773183. doi: 10.3389/fpubh.2026.1773183 (PMC13017915; doi:10.3389/fpubh.2026.1773183)
Supplement: Supplementary file 13 [file Table_13.docx]

**Supplementary File 13 Homogeneity test of the initial scale items** **(n = 113)**

| Item code | Cronbach’s Alpha if item deleted | Communality | Factor loading |
| --- | --- | --- | --- |
| C1 | 0.93 | 0.04 | - |
| C2 | 0.93 | 0.04 | - |
| C3 | 0.92 | 0.40 | 0.63 |
| C4 | 0.92 | 0.27 | 0.52 |
| C5 | 0.92 | 0.49 | 0.70 |
| C6 | 0.92 | 0.43 | 0.65 |
| C7 | 0.92 | 0.34 | 0.58 |
| C9 | 0.91 | 0.65 | 0.80 |
| C11 | 0.92 | 0.54 | 0.73 |
| C12 | 0.92 | 0.54 | 0.73 |
| C13 | 0.92 | 0.61 | 0.78 |
| C14 | 0.91 | 0.66 | 0.81 |
| C15 | 0.91 | 0.60 | 0.77 |
| C16 | 0.92 | 0.40 | 0.64 |
| C17 | 0.92 | 0.53 | 0.73 |
| C18 | 0.92 | 0.42 | 0.64 |
| C19 | 0.92 | 0.19 | - |
| C20 | 0.92 | 0.61 | 0.78 |
| C21 | 0.92 | 0.63 | 0.79 |
| C22 | 0.92 | 0.59 | 0.77 |
