# Supplementary material for: Development and validation of the quiet quitting behavior scale: a mixed-methods study with primary healthcare workers in China
Source: Front Public Health. 2026 Mar 12;14:1773183. doi: 10.3389/fpubh.2026.1773183 (PMC13017915; doi:10.3389/fpubh.2026.1773183)
Supplement: Supplementary file 14 [file Table_14.DOCX]

**Supplementary File 14 Demographic characteristics of primary medical staff in the formal survey (n=407)**

| **Variable** | **Category** | **n** | **%** |
| --- | --- | --- | --- |
| Gender | Male | 138 | 33.91 |
|  | Female | 269 | 66.09 |
| Age | ≤25 years | 57 | 14.00 |
|  | 26–35 | 141 | 34.64 |
|  | 36–45 | 105 | 25.80 |
|  | 46–55 | 94 | 23.10 |
|  | >55 years | 10 | 2.46 |
| Marital Status | Married | 300 | 73.71 |
|  | Unmarried | 86 | 21.13 |
|  | Divorced | 10 | 2.46 |
|  | Other | 11 | 2.70 |
| Years of Work | ≤5 years | 110 | 27.03 |
|  | 6–10 years | 96 | 23.59 |
|  | 11–15 years | 62 | 15.23 |
|  | 16–20 years | 30 | 7.37 |
|  | >20 years | 109 | 26.78 |
| Education Level | Junior high school or below | 2 | 0.49 |
|  | High school or secondary school | 65 | 15.97 |
|  | Associate Degree | 131 | 32.19 |
|  | Bachelor’s Degree | 207 | 50.86 |
|  | Master’s or above | 2 | 0.49 |
| Professional Title | None | 81 | 19.90 |
|  | Junior | 158 | 38.82 |
|  | Intermediate | 132 | 32.43 |
|  | Senior | 36 | 8.85 |
| Monthly Income (RMB) | <2,000 | 16 | 3.93 |
|  | 2,000–3,000 | 79 | 19.41 |
|  | 3,001–4,000 | 91 | 22.36 |
|  | 4,001–5,000 | 73 | 17.94 |
|  | >5,000 | 148 | 36.36 |
| Total |  | 407 | 100.00 |
